# Relationship between Cefquinome PK/PD Parameters and Emergence of Resistance of *Staphylococcus aureus* in Rabbit Tissue-Cage Infection Model

**DOI:** 10.3389/fmicb.2016.00874

**Published:** 2016-06-07

**Authors:** Mingpeng Xiong, Xun Wu, Xiaomei Ye, Longfei Zhang, Shuyi Zeng, Zilong Huang, Yuzhi Wu, Jian Sun, Huanzhong Ding

**Affiliations:** National Risk Assessment Laboratory for Antimicrobial Resistance of Microorganisms of Animal Original Bacteria, College of Veterinary Medicine, South China Agricultural UniversityGuangzhou, China

**Keywords:** *Staphylococcus aureus*, cefquinome, antimicrobial resistance, rabbit tissue-cage infection model, PK/PD parameters

## Abstract

In order to explore the relationship between different antibiotic dosing regimens and selective enrichment of resistant strains, tissue-cage infection model was established in rabbits to study relationship between cefquinome pharmacokinetic/pharmacodynamic parameters and the change of susceptibility of *Staphylococcus aureus* (*S. aureus*). In this model, above 10^8^ CFU/mL of *S. aureus* culture were exposed to cefquinome concentrations below the MIC_99_ (the minimal concentration that inhibits colony formation by 99% *in vitro*, 0.3 μg/mL), between the MIC_99_ and the MPC (the mutant prevent concentration *in vitro*, 1.6 μg/mL), and above the MPC after intramuscular injection with cefquinome at doses of 4, 8, 16, and 32 mg/kg of body weight (bw) once daily for 5 days or 4, 8, 16, and 24 mg/kg of bw twice daily for 2.5 days. Samples of tissue-cage fluid were collected from the tissue-cage at 2, 4, 6, 8, 10, 12, 24 h after each dosing (one dosing daily) or at 2, 4, 6, 8, 10, and 12 h (two dosing daily). Cefquinome concentration, susceptibility of *S. aureus* to cefquinome, and bacterial numbers at the infected site were monitored. The MICs of *S. aureus* and the fraction of resistant bacteria both increased when cefquinome concentrations fluctuated between the MIC_99_ and MPC. Resistant bacteria were selected *in vivo* when %*T* > MPC was < 58% of administration interval or %*T* > MIC_99_ was ≥70% of administration interval. These findings demonstrate that low-level, cefquinome-resistant *S. aureus* were selected predominantly when drug concentrations fell inside a concentration window in *in vivo* model, which was evidenced by pulsed-field gel electrophoresis. The selection of resistant bacteria arose from both susceptible bacteria being killed and resistant bacteria re-growth. Keeping drug concentrations above the MPC for ≥58% of administration interval provides a strategy to achieve effective antibacterial activity and minimize the emergence of resistance to cefquinome.

## Introduction

Cephalosporins, which are among the most important drugs, are used to combat bacterial infections including those caused by *S. aureus* in humans and animals. Cefquinome is a fourth-generation cephalosporin, which was only used in veterinary applications and approved for the treatment of acute mastitis caused by *S. aureus* (Committee for Veterinary Medicinal Products [CVMP], 2009). However, due to the overuse and abuse of animal drugs, the selective enrichment of resistant strains becomes a serious problem worldwide (Howard et al., 2013) and will be a great threat to human health. In order to prevent or lessen the emergence of resistant bacteria, it is of great importance to explore the mechanism of selected resistant strains under different dosing regimens and then optimize dosing regimen.

Appropriate antibiotic dosing regimen is not only the key to the eradication of infection-causing bacteria but also has an important role in inhibiting the emergence and proliferation of antibiotic-resistant strains (Toutain et al., 2002). One of the approaches to restrict the acquisition of resistance is the mutant selection window (MSW) hypothesis (Zhao and Drlica, 2001). The hypothesis offered a range of drug concentrations: the lower boundary is MIC_99_ (the minimal concentration that inhibits colony formation by 99%) and the upper boundary is MPC (the mutant prevention concentration that inhibits growth of the least-susceptible mutant subpopulation). Maintaining concentrations outside the MSW can prevent the selection of resistant bacteria. In addition, MIC- and MPC-related pharmacokinetic/pharmacodynamic (PK/PD) parameters play an important role in the application of the MSW hypothesis to elucidate the development of resistance (Firsov et al., 2003). The tissue-cage model was usually used to study the PK/PD parameters because drug concentrations and the selective amplification of resistant bacteria can be directly measured at the infected site, and the *in vivo* model also simulates the dynamic relationship among the antimicrobial agents, organism, and pathogens (Craig, 1993; Fernandez et al., 1999).

In this study, a standard *S. aureus* was exposed to different cefquinome dosing regimens in rabbit tissue-cage infection model. Additionally, we examined the relationship between the resistant strains and the original susceptible strain by pulsed-field gel electrophoresis (PFGE). The purpose of the study was to validate that resistant bacteria would be selected and enriched predominantly when drug concentrations fell inside a concentration window (MSW), the boundaries of which determined by the agar dilution method.

## Materials and Methods

### Antimicrobials and Chemicals

Cefquinome standard was provided by China Institute of Veterinary Drugs Control (Beijing, PR China). Cefquinome sulfate injection (Cobactan, the Batch Number A659A01) was obtained from MSD Animal Health (Shanghai) Trading Co. Ltd., Penicillin, as a sodium salt for injection, was purchased from Jia Tai Animal Pharmaceutical Co, Ltd., (Sichuan, China). Xylazine hydrochloride injection was purchased from Shengda Animal Pharmaceutical Co. Ltd (Jilin, China). Ketamine hydrochloride injection was purchased from Hengrui Medicine Co. Ltd (Jiangsu, China).

### Bacterial Strain and Susceptibility Testing

*S. aureus* ATCC 29213 was selected for the study. MIC of cefquinome was determined by means of the agar dilution method according to the Clinical and Laboratory Standards Institute (CLSI) reference method (Clinical and Laboratory Standards Institute [CLSI], 2013). The MIC of the strain in the blank tissue-cage fluid was further measured by the broth micro-dilution method. MIC_99_ and MPCs were determined using a previously method (Lu et al., 2003). Briefly, for MIC_99_, serial dilutions of stationary-phase cultures were applied to Mueller–Hinton (MH) agar plates containing various drug concentrations. After incubation at 37°C for 24 h, bacterial colonies were counted. The minimal concentration that inhibits growth of 99% colonies was defined as MIC_99_.

For MPCs, series of MH agar plates containing known drug concentrations were inoculated with above 10^10^ CFU *S. aureus.* The inoculated plates were incubated at 37°C for 72 h and screened visually for growth. The preliminary MPC was taken as the lowest drug concentration that prevented growth. To estimate the exact MPC, logarithms of bacterial numbers were plotted against antibiotic concentrations. The exact MPC was taken as the point that intersected the theoretical limit of detection.

### Rabbit Tissue-Cage Infection Model

Thirty-eight New Zealand White rabbits, three- to four-month-old, weighing 2.5–3.2 kg, were supplied by Guangzhou NanFang Medical Experimental Animal Science & Technology Development Co. Ltd. They were kept individually in cages and fed antibiotic-free food and water. The experimental protocol was approved by the Committee on the Ethics of Animals of South China Agricultural University (Approval number 2015-04; March 11, 2015). Rabbits were anesthetized by use of xylazine (4 mg/kg) and ketamine (40 mg/kg) intramuscularly (Cui et al., 2006). Then a 5-cm incision was made in the dorsal middle of each rabbit and an autoclaved plastic ball (Wiffle ball, 43 mm in diameter, with a volume of 34 mL) was implanted subcutaneously into each rabbit through the incision. The incision was closed with sutures. After the surgery, the rabbits were injected with penicillin intramuscularly (100,000 IU/kg) twice daily for 3 days to prevent infection. About 4–6 weeks after implantation, the Wiffle ball had been wrapped in a layer of connective tissue and was full of clear, bright tissue-cage fluid. About 0.5 mL tissue-cage fluid was collected from each plastic ball to confirm sterile tissue-cage fluid. More than 10^10^ CFU of *S. aureus* culture in the logarithmic growth phase were concentrated in 2 mL of 0.9% NaCl solution and injected into each plastic ball. Two days after infection, 0.5 mL tissue-cage fluid was removed from each plastic ball for bacteria counting. Rabbits having above 10^8^ CFU/mL bacterial concentration in tissue-cage fluid were treated with cefquinome.

### Dosing Regimens and Pharmacokinetic Measurements

According to the results of the preliminary experiments, rabbits were administered cefquinome at doses of 4, 8, 16, and 32 mg/kg of bw once daily for 5 days or 4, 8, 16, and 24 mg/kg of bw twice daily for 2.5 days. For treatment groups, cefquinome sulfate injection was administrated intramuscularly, beginning at day 3 after infection. Control groups were injected with sterile normal saline in the same way. About 0.5 mL sample of tissue-cage fluid was collected from the plastic ball with a syringe at 2, 4, 6, 8, 10, 12, and 24 h after each dosing for 24 h dosing intervals. For groups with 12 h dosing intervals, samples were collected at 2, 4, 6, 8, 10, and 12 h after each dosing. Samples of tissue-cage fluid were clarified by centrifugation at 8900 ×*g* for 10 min and stored at -20°C until analyzed.

Cefquinome concentrations in tissue-cage fluid were determined by HPLC-MS/MS (Agilent Technologies, USA; Zhang B.X. et al., 2014). Briefly, after thawing, each sample (200 μL) including blank sample was added to the same volume acetonitrile for deproteinization, and then was clarified by centrifugation. Two-hundred microliters clear supernatant and 800 μL water were mixed and then transferred to an HPLC-MS/MS vial. A calibration curve was made by adding a known amount of cefquinome to blank tissue-cage fluid over a concentration range of 0.005–3.000 μg/mL. All assays were made in triplicate and the correlation coefficient for the calibration curves were always ≥0.99.

### Quantification of the Antimicrobial Effect and Susceptibility Changes of *S. aureus* to Cefquinome

Multiple samples (1 mL) were collected from the Wiffle ball in each rabbit daily before, during the treatment (after every administration), and 24 and 48 h after the termination of treatment for one dosing daily groups, or 12 and 24 h for two dosing daily group. To quantify the antimicrobial effect and the mutant fraction, each sample (0.6 mL) was serially diluted with the sterile normal saline and applied to MH agar either lacking drug or containing cefquinome at 1 × MIC of cefquinome. After incubation for 24–48 h, colonies were calculated. The fraction of mutant (%) was determined daily as the ratio of number of colonies grown on 1 × MIC cefquinome-containing MH agar to the number grown on drug-free agar. The low limit of accurate detection was 200 CFU/mL. Other sample (0.4 mL) was incubated overnight in drug-free MH broth and then the MIC was determined as described above.

### Resistant Strain Analysis

Resistant strains were chosen randomly from the 1 × MIC of cefquinome-containing MH agar, and then passaged five times on drug-free high salt mannitol medium agar. The MICs of resistant strains were determined as described above. Eight randomly selected resistant strains and one original susceptible strain (bacteria were injected into Wiffle ball in the beginning) were characterized by PFGE using the CHEF-MAPPER System (Bio-Rad Laboratories, Hercules, CA, USA), as described previously (McDougal et al., 2003). The PFGE patterns were analyzed with BioNumerics software (Applied Maths, Sint-Martens-Latem, Belgium) using the Dice similarity coefficient with a cut-off at 90% of the similarity values to indicate identical PFGE types.

### Date Analysis

Pharmacokinetic parameters such as *C*_max_ (the maximum concentration) and AUC_0-24__h_ or AUC_0-12__h_ (24 or 12 h area under the concentration–time curve) were calculated for each tissue-cage, from 0 to 12 h or from 0 to 24 h after every drug administration. AUC_0-24__h_ or AUC_0-12__h_ was calculated by the trapezoidal rule. PK/PD parameters such as %*T* > MIC_99_ (the percentage of time that drug concentration remains above the MIC_99_), %*T* > MPC (the percentage of time that drug concentration remains above the MPC), *C*_max_/MIC_99_, *C*_max_/MPC were calculated by using WinNonlin program (version 5.2, Pharsight Corporation, Mountain View, CA, USA).

Fisher’s exact text was used for statistical analysis of the PK/PD dates, with two groups of six infected but untreated rabbits as a control. *P* values < 0.05 were considered statistically significant.

## Results

### MIC, MIC_99_, and MPC of Cefquinome for *S. aureus*

The MICs of cefquinome against *S. aureus* ATCC 29213 on the agar plates and in the blank tissue-cage fluid were both 0.5 μg/mL. The cefquinome MIC_99_ was 0.3 μg/mL. The preliminary MPC of cefquinome and the exact MPC of cefquinome were 2 and 1.6 μg/mL, respectively. All experiments were performed in triplicate on different occasions.

### The Pharmacokinetics of Cefquinome

**FIGURE 1:**
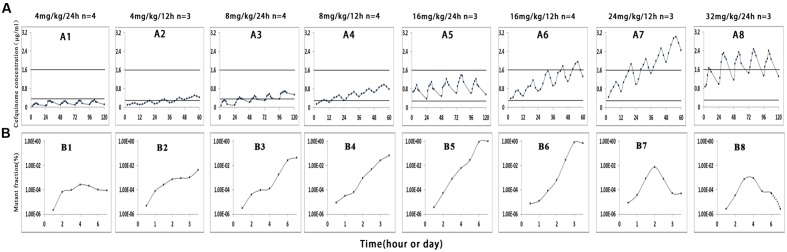
**Effect of cefquinome concentration on mutant enrichment. 1, 3, 5, and 8 correspond to the four different treatment groups once daily for 5 days, 2, 4, 6, and 7 correspond to the four different treatment groups twice daily for 2.5 days, *n* is the animal number per group.** *In vivo* simulated pharmacokinetics **(A)** and the fraction of resistant mutant **(B)**. Since the amount of bacteria for the group 32 mg/kg/24 h was below the minimum detection limit (200 CFU/mL) on the seventh day (**Figure 2**), we cannot draw the mutant fraction (shown in dotted line).

Cefquinome concentrations in each tissue-cage of treatment groups were determined and divided into eight groups as shown in **Figure 1** (A1–A8). Cefquinome concentrations shown in each group were the mean values of concentrations from every rabbit tissue-cage. The mean concentrations located in different positions in the MSW: almost completely below the lower window boundary (MIC_99_) (A1), across the MIC_99_ (A2–A4), completely inside the MSW (A5) and across the upper window boundary (MPC) (A6–A8). The mean values of AUC_0-12__h_ or UC_0-24__h_ and *C*_max_ following multiple dosing are shown in **Table 1**. The AUCs from 0 to 24 h and from 0 to 12 h after every administration, as determined by trapezoidal rules, ranged from 5.590 to 69.782 μg⋅h/mL and from 6.874 to 55.558 μg⋅h/mL, respectively. The maximum concentrations (*C*_max_) were calculated by WinNonlin program, ranging from 0.231 to 2.154 μg/mL and from 0.308 to 1.954 μg/mL, respectively.

**Table 1:** The pharmacokinetic parameters of cefquinome following multiple dose in rabbit tissue-cage infection model.

Cefquinome dose (mg/kg of body weight)	24 h intervals		12 h intervals	
	
	AUC_0-24__h_ (μg⋅h/mL)	*C*_max_ (μg/mL)	AUC_0-12__h_ (μg⋅h/mL)	*C*_max_ (μg/mL)
4	5.590	0.231	6.874	0.308
8	18.398	0.454	16.468	0.618
16	30.846	1.091	22.070	1.304
24	ND	ND	55.558	1.954
32	69.782	2.154	ND	ND

*The AUC_0-24__h_, AUC_0-12__h_, and C_max_ were the mean values of multiple dose. ND, not done.*

### The Antimicrobial Effect and Susceptibility Changes

**FIGURE 2:**
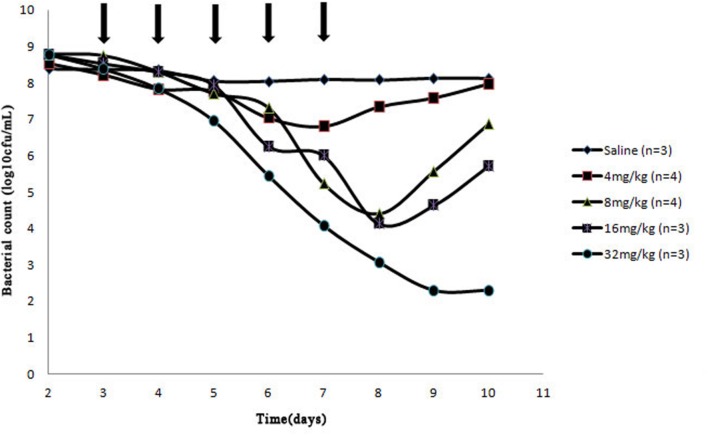
**Effect of cefquinome at different doses on bacterial survival in the tissue-cage model (24 h intervals).** Three days after infection, different doses (0, 4, 8, 16, and 32 mg/kg of body weight by use of intragluteal injection) of cefquinome were administered once daily for 5 days (indicated by the arrow). *n* is the animal number per group.

**FIGURE 3:**
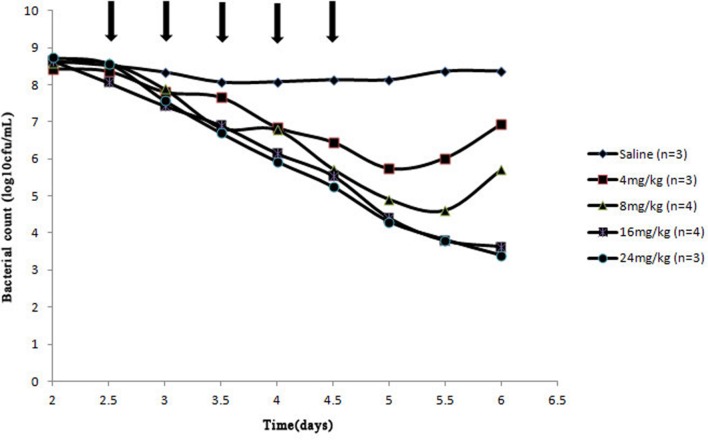
**Effect of cefquinome at different doses on bacterial survival in the tissue-cage model (12 h intervals).** Three days after infection, different doses (0, 4, 8, 16, and 24 mg/kg of body weight by use of intragluteal injection) of cefquinome were administered twice daily for 2.5 days (indicated by the arrow). *n* is the animal number per group.

After a Wiffle ball was implanted subcutaneously into each rabbit for a week, 4 of 38 rabbits died of infection. For the survivors, no serious illness or distress was observed during the entire experimental period when above 10^10^ CFU bacteria were injected into the implanted ball. The effects of cefquinome on bacterial survival in the tissue-cage model are shown in **Figures 2** and **3**. Bacterial concentration remained constant (about 10^8^ CFU/mL) when rabbits were treated with sterile normal saline for five times. Compared to the two saline groups, growth inhibition of *S. aureus* was observed in groups at the dose of 4 mg/kg with 24 h intervals, bacterial numbers decreased slightly; for dose at 4, 8 mg/kg with 12 h intervals and at 8, 16 mg/kg with 24 h intervals, bacterial numbers decreased obviously during the treatment, but bacterial re-growth was observed after the termination of treatment; for groups at the dose of 32 mg/kg with 24 h intervals and 16, 24 mg/kg with 12 h intervals, bacterial numbers decreased significantly and no bacterial re-growth was observed during the whole observation period. Compared to the groups with one dosing daily, bacterial numbers decreased more at the same dose in the same time in the groups with two dosing daily. The reason is that cefquinome concentration in the tissue-cage rose more in the same time. For example, the bacterial numbers were about 10^5^ CFU/mL on the fifth day and the highest concentration of cefquinome was close to 0.8 μg/mL in the group of 8 mg/kg with two dosing daily, but the bacterial numbers were about 10^7^ CFU/mL and the highest concentration of cefquinome was close to 0.3 μg/mL in the group of 8 mg/kg with one dosing daily.

Samples of *S. aureus* in tissue-cage fluid were checked for susceptibility changes and mutant fraction after each treatment. When drug concentrations were mostly below the MIC_99_ (**Figure 1**, Panel A1 and A2) or exceeded the MPC (**Figure 1**, A8), no loss in susceptibility was observed, but mutant fraction rose slightly and then remained unchanged (**Figure 1**, B1 and B2) or decreased (**Figure 1**, B8). When drug concentration crossed the MIC_99_ (**Figure 1**, A2–A4), completely fell inside the MSW (**Figure 1**, A5), or crossed the MPC (**Figure 1**, A6 and A7), the MICs increased to 1–2 μg/mL after the third or fourth dose and the resistant strains were readily selected. The detailed relationship between values of PK/PD parameters and selection of resistance is shown in **Table 2**.

**Table 2:** Correlation of PK/PD parameters with selection of resistance.

PK/PD parameter value	Groups	Fraction of rabbits with resistance bacteria	*P*
**%*T* > MIC_99_**			
< 70%	A1, A2	0/7	NA
≥ 70%	A3–A8	11/17	0.009
**%*T* > MPC**			
< 55% 55–58%	A1–A6 A7	20/22 1/3	7.43 × 10^-5^ 0.33
≥58%	A8	0/3	NA
**AUC/MIC_99_ (h)**			
<61.33	A1, A2	0/7	NA
61.33–185.19	A3–A7	11/18	0.013
>185.19	A8	0/3	NA
**AUC/MPC (h)**			
<11.5	A1, A2	0/7	NA
11.50–34.72	A3–A7	11/18	0.013
>34.72	A8	0/3	NA
***C*_max_/MIC_99_**			
<1.51	A1, A2	0/7	NA
1.51–6.51	A3–A6	13/15	0.0005
≥6.51	A7, A8	1/6	0.5
***C*_max_/MPC**			
<0.28	A1, A2	0/7	NA
0.28–1.22	A3–A6	13/15	0.0005
≥1.22	A7, A8	1/6	0.5

*All parameters were calculated using total drug concentrations. NA, not applicable. P values were calculated by Fisher’s exact test, with a set of six infected but untreated rabbits used as a control, high values indicate no difference with the control.*

### PK/PD Parameters and the Emergence of Resistance

The correlation of MIC- and MPC-related PK/PD parameters with the selection of resistance was displayed in **Table 2**. %*T* > MIC_99_ is not only the main pharmacodynamic parameter to measure the bactericidal activity of time-dependent antibiotics but also the common parameter to restrict mutant enrichment (Craig, 2007). Cefquinome is known as time-dependent antibiotic. In all seven tissue-cages, when %*T* > MIC_99_ was < 70%, there was no loss of susceptibility. But, loss of bacterial susceptibility occurred in 11 of 17 tissue-cages when %*T* > MIC_99_ ≥ 70%. So %*T* > MPC is also the suitable parameter with restricting mutant enrichment. The resistant strains were selected in 20 of 22 tissue-cages when %*T* > MPC was < 55%.

In addition, other PK/PD parameters also indicated statistically significant correlations with the selection of resistance (**Table 2**). Mutant selection was promoted when AUC/MIC_99_ fell between 61.33 h and 185.19 h or AUC/MPC fell between 11.50 h and 34.72 h. In other forms, the MSWs extended from 1.51 to 6.51 and from 0.28 to 1.22, respectively, when *C*_max_/MIC_99_ and *C*_max_/MPC were considered.

### Analysis of Resistant Strains

Nine *S. aureus* strains were determined by PFGE. A dendrogram of percent similarity, calculated with Dice coefficients from the PFGE data using a cut-off of 90%, revealed one major cluster of strains. It means that the original susceptible strain (*S. aureus* ATCC 29213) and the resistant strains were identical PFGE types. Clearly, the resistant strains originated from the initial population. The cefquinome MICs for resistant strains were both 1–2 μg/mL.

## Discussion

According to the MSW hypothesis, pathogens exposed to continuous drugs would evolve into resistant bacteria if drug concentrations fell inside the MSW (Zhao and Drlica, 2001). In this experiment, when the drug partly or wholly dropped into the MSW, the fraction of mutants obviously increased (**Figure 1**, B3–B6). The study proved that the boundaries of MSW determined by *in vitro* agar plates or blank tissue-cage fluid were basically consistent with those observed in *in vivo* model. It is also in agreement with other studies (Croisier et al., 2004; Cui et al., 2006; Almeida et al., 2007; Bourgeois-Nicolaos et al., 2007; Zhu et al., 2012; Ni et al., 2014; Zhang B. et al., 2014). However, Mei et al. (2015) found that the emergence of fosfomycin-resistant mutations was found *in vitro* but not *in vivo* by using rabbit infection model when the drug concentration fell into the MSW.

From **Figure 1** (B3–B6), **Figures 2** and **3**, we found that bacterial numbers gradually decreased (except for 4 mg/kg with 24 h intervals) during the treatment (after every administration), but the mutant fraction gradually increased, indicating the resistant bacteria were gradually enriched and susceptible bacteria partially were killed. After the termination of treatment, the mutant fraction of one group on the sixth day is 100% (**Figure 1**, B5) and the total amount of bacteria gradually increased (**Figure 2**), indicating that bacteria in this group were all resistant stains and they also grew gradually. Thus, the selection of resistant bacteria for cefquinome arose from both susceptible bacteria being killed and resistant bacteria re-growth.

Antimicrobial PK/PD parameters have been used to assess the clinical effects and their potential in the prevention of antibiotic resistance development (Leroy et al., 2012). In our study, %*T* > MIC_99_ and %*T* > MPC ratios that protect against the selection of resistant bacteria were < 70 and ≥ 58%, respectively. Wang et al. (2014) found that %*T* > MIC target to produce a bacteriostatic effect was 40–50% for cefquinome against *S. aureus* in a Neutropenic Mouse Thigh Model. The possible reasons for the different between the above two studies may be that the different models were used and the latter did not consider the emergence of resistant bacteria. Our result is similar to Zhang B. et al. (2014), since they defined a %*T* > MPC ratio of ≥ 50% to prevent emergence of cefquinome-resistant *Escherichia coli* in piglet tissue-cage model. Some investigators reported that %*T* > MIC_99_ was the most-predictive parameter for the time-dependent drugs for resistance suppression and AUC/MIC_99_ was the most-predictive parameters for the concentration-dependent drugs for resistance suppression in animal models (Jumbe et al., 2003; Knudsen et al., 2003; Tam et al., 2009; Wang et al., 2014). However, other investigators found that antimicrobial–pathogen combinations in animal models, such as cefquinome against *Escherichia coli*, vancomycin against *S. aureus*, linezolid against *Enterococcus faecalis* (Bourgeois-Nicolaos et al., 2007; Zhu et al., 2012; Zhang B. et al., 2014), still cannot reach a conclusion that which pharmacodynamic parameters is the most-predictive parameter. Our work also did not identify the most-predictive parameter correlating with the resistance because only two dosing frequencies were used. Because %*T* > MIC_99_ < 70% cannot keep an effective antibacterial activity (**Table 2**, **Figures 2** and **3**) and cefquinome is the time-dependent drug, %*T* > MPC may be the best parameter for achieving an effective antimicrobial activity and resistance suppression.

In this study, bacterial numbers decreased slightly for the dose of 4 mg/kg at 12 h intervals or 24 h intervals, and bacterial re-growth was observed after the termination of treatment, suggesting 4 mg/kg maybe not sufficient to combat infection. Intramuscular administration of cefquinome at the dose of 8–32 mg/kg bw exhibited excellent bactericidal effect against *S. aureus* in tissue cage. However, due to the high MPC value of cefquinome against *S. aureus* (1.6 μg/ml), concentrations generated by those doses fell within the MSW, and dramatic increases in the mutant fraction occurred in the treatment group of 8–16 mg/kg bw. Although no resistant mutant was observed for 32 mg/kg group, the dose seems to be too high, compared to recommended dose of 1–3 mg/kg in veterinary clinic. Considering the relevance for actual treatment, a strategy is to keep %*T* > MPC ≥ 58% of the interval at the infected site of the target animal for cefquinome against *S. aureus*. If the doses exceeding MPC are unacceptably high, shorter intervals such as 6 h or 8 h could be applied. Since the pharmacokinetic profiles of cefquinome may vary in different animals, the optimal dosage regimen must integrate pharmacokinetic parameters and pharmacodynamic parameters from specific animal species. Of course, other cefalosporins with narrow MSW may also be a good choice.

Since we cannot guarantee the entire operation in a sterile environment during animal experiment, it is necessary to identify species relationship the original susceptible strain and the selected resistant strains by PFGE. The results demonstrated that these resistant bacteria originated from the cefquinome-susceptible *S. aureus* population present in the site of local infection.

In Gram-positive bacteria such as *S. aureus*, resistance to β-lactam antibiotics occurs majorly as a result of modification of penicillin-binding proteins (PBPs), with β-lactamase production as a minor way. Since methicillin-resistant *S. aureus* (MRSA) appeared at the start of the 1960s (Jevons et al., 1963), MRSA has been the primary resistant in *S. aureus*. Because MRSA is not only β-lactamase-mediated resistant bacteria, but also contained a unique PBP (PBP 2a). The gene encoding PBP2a is mecA and lies in a specificmobile genetic element called the staphylococcal cassette chromosome mec (SCCmec) that integrates into the chromosome using recombinases (ccrAB or ccrC) carried on the SCCmec element itself (Wang and Archer, 2010). However, expression of methicillin resistance also associated with some other factors, such as the FEM (factor essential for methicillin resistance) or auxiliary factors (Komatsuzawa et al., 1999). Although these mechanisms have already been discovered, the mechanisms of cephalosporin against *S. aureus* are still very complex. In the next experiment, we will carry out further study to explore mutant genes causing the acquired cefquinome-resistance.

In order to extend similar studies to other antimicrobial–pathogen combinations, our study suggests that careful attention should be paid to population size, experiment duration, the choice of dosage, and the clear mechanism of drug resistance. The mutants derived from wild-type bacteria was very low (10^-6^–10^-8^; Drlica, 2001), when bacteria were exposed to drug, the bacterial number must be sufficient large (e.g., > 10^8^ CFU/mL) to ensure that resistant bacteria emerged. Experiment must have sufficiently duration to allow for the emergence of resistant bacteria since their appearance may be delayed if there is a fitness cost associated with resistance (Andersson and Levin, 1999). Drug concentrations by the appropriate dose should fall into the different positions of MSW (including outside MSW) as far as possible. There must be a clear mechanism of drug resistance to determine whether the resistant bacteria were caused by the pressure effect of the drug selection.

We could conclude that the date obtained with cefquinome as the time-dependent drug against *S. aureus* is in support of the MSW hypothesis. Keeping drug concentrations above the MPC for ≥58% of administration interval provides a strategy to keep effective antibacterial activity and minimize the emergence of resistance.

## Author Contributions

HD and MX: designed and conducted the experiment. XW, XM, and LF: animal management, health and welfare. MX, SZ, ZH, and YW: collected and analysis. MX, JS: analysis date. MX drafted the manuscript. All authors read and approved the final manuscript.

## Conflict of Interest Statement

The authors declare that the research was conducted in the absence of any commercial or financial relationships that could be construed as a potential conflict of interest.
